# Synthesis and Antibacterial Activity of Novel Hydroxy Semicarbazone Derivatives

**Published:** 2016

**Authors:** Elham Hariri, Arash Mahboubi, Mohammad Fathi, Parisa Rahmani, Kamaleddin Haj Mohammad Ebrahim Tehrani, Mohammad Babaeian, Vida Mashayekhi, Farzad Kobarfard

**Affiliations:** a *Department of Medicinal Chemistry, School of Pharmacy, Phytochemistry Research Center, Shahid Beheshti University of Medical Sciences, Iran. *; b *Department of Pharmaceutics, School of Pharmacy, Shahid Beheshti University of Medical Sciences, Iran.*; c *Department of Anestesiology, Shahid Modarres Hospital, Shahid Beheshti University of Medical Sciences, Tehran, Iran.*

**Keywords:** *N*-hydroxy semicarbazone, Antibacterial, Broth microdilution assay, Ketones, Hydrophilicity

## Abstract

A series of hydroxyl semicarbazone derivatives of substituted diaryl ketones and acetophenones were synthesized and their structures were confirmed by analytical and spectroscopic methods including elemental analysis, infrared and nuclear magnetic resonance spectroscopy. The derivatives were prepared by a condensation reaction between *N*-hydroxy semicarbazide and substituted diaryl ketones or acetophenones leading to the desired hydroxysemicarbazones with excellent purity. The synthesized hydrazones were then evaluated for their inhibitory activity against bacterial strains including *S. aureus*, *E. Coli*, *P. aeruginosa*, *K. pneumonia* and *M. luteus*. Among the tested derivatives, compounds 2, 6 and 7 exhibited the highest bioactivity. Analysis of the activity data suggests that hydrophilicity is an important factor for the bioactivity of compounds 2 and 6 and also their selectivity over the gram-negative bacteria.

## Introduction

**Figure 1:**
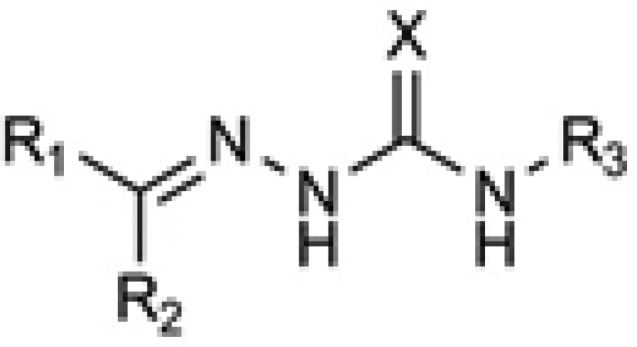
General structure of active (thio) semicarbazones. X = O, S; R = various aromatic and aliphatic substituents

In the drug discovery efforts to combat against microbial infections, (thio) semicarbazones have attracted considerable attentions and many derivatives of this class have been reported to possess promising bioactivity. In addition some (thio) semicarbazones have been marketed in some periods as antimicrobial drugs and some more are under investigations as potential ones. Because of their interference with vital biochemical processes in the living cells such as deoxyribonucleotide synthesis (1), cell wall biosynthesis (2) and maintaining thiol contents (3), (thio)semicarbazones have been reported to exert antibacterial (4), antimycobacterial (5), anticancer (1), antifungal (6) and antimalarial (7) activities. The general structure of the active (thio) semicarbazones is disclosed in Figure 1, which consists of an aromatic system linked to the (thio) semicarbazone moiety. Working on this structural backbone, many research projects have been conducted to discover novel (thio) semicarbazone derivatives with optimized potency and safety profiles. 

**Figure 2:**
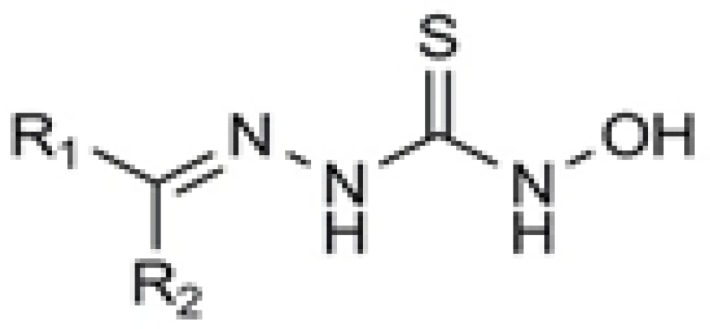
Antimycobacterial *N*-hydroxy semicarbazone derivatives reported by Sriram *et al*. (8).

In a research work carried out by Sriram and his colleagues, some *N*-hydroxythiosemicarbazones of different aromatic systems were synthesized and tested for their antimycobacterial activity (8). Among the employed aromatic carbonyl compounds to condense with *N*-hydroxythiosemicarbazide, the order of activity of the tested compounds was found to be Schiff bases of diaryl ketones > acetophenones > aromatic aldehydes (Figure 2). 

**Figure 3:**
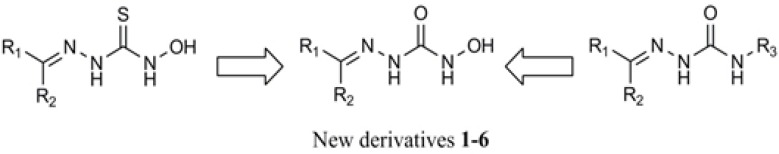
Design of the novel *N*-hydroxy semicarbazone derivatives based on the structural elements of active *N*-hydroxy thiosemicarbazones (left) and semicarbazones (right

Considering the above findings and in continuation of our interest to study the bioactivity of hydrazone derivatives (9-12), in the present work we describe the synthesis and antibacterial activity of some *N*-hydroxysemicarbazones of diaryl ketones and substituted acetophenones. These derivatives could be considered as bioisoesters of active *N*-hydroxythiosemicarbazones in which the thiocarbonyl is substitutes by carbonyl functional group. The new derivatives could also be viewed as active antimicrobial semicarbazones bearing a hydroxyl substitutent at their thioamide nitrogen (Figure 3).

## Experimental

*General*

Melting points were measured using an Electrothermal 9100 apparatus and are uncorrected. The Infrared spectra were obtained with a Perkin-Elmer 843 spectrometer. Proton nuclear magnetic resonance (^1^H NMR) and carbon nuclear magnetic resonance (^13^C NMR) spectra were determined by a Bruker Avance DRX 500 MHz spectrometer and the samples were dissolved in DMSO-*d*_6 _and tetramethylsilane (0.05% v/v) as internal standard. All the compounds were analyzed for C, H and N on a Costech model 4010 and agreed with the proposed structures within ±0.4% of the theoretical values. Hydroxysemicarbazide (13) and 4,4’-dihydroxybenzophenone 2,4-dinitrophenylhydrazone (compound **7**) (13) were prepared according to previously reported methodologies.

*Benzophenone hydroxysemicarbazone (1)*

*Method A. (conventional synthesis)*

Benzophenone (3.64 g, 20 mmol) was added to 50 mL of absolute ethanol and the mixture was heated at 70 °C. Then, a solution of hydroxysemicarbazide (1.82 g, 20 mmol) in 20 mL of water and 7 drops of glacial acetic acid was added dropwise to the solution via a dropping funnel. The mixture was heated under reflux for 48 h and then concentrated by vacuum distillation. The resulting precipitate was filtered and recrystallized from methanol to afford the title compound as white powder (1.27 g, 24%).

*Method B. (microwave-assisted synthesis)*

In a 100 mL beaker, benzophenone (3.64 g, 20 mmol) was dissolved in 50 mL of absolute ethanol by a gentle heating. A solution of hydroxysemicarbazide (1.82 g, 20 mmol) in 20 mL of water and 7 drops of glacial acetic acid was then added in three portions to the benzophenone solution. After addition of each portion, the beaker was placed in a microwave reactor set at 600 w for five 30-second periods. After the completion of the procedure, the beaker was cooled at room temperature and the resulting precipitate was filtered. The crude was recrystallized from 2-propanol to afford the title compound.

White powder (3.31 g, 65%): mp 162.5-165‎ °C; IR (KBr): 3200 (OH), 3350 (NH), 1668 (C=O); ^1^H NMR (CDCl_3_/500 MHz): *δ* 8.30 (br s, 1H), 7.85 (br s, 1H), 7.65-7.36 (m, 5H, Ar H), 7.21-7.17 (m, 3H, Ar H), 7.12 (d, *J*=8.15, Ar H); Anal. Calcd for C_14_H_13_N_3_O_2_ (255.27): C, 65.87; H, 5.13; N, 16.46. Found: C, 65.71; H, 5.14; N, 16.50.

*Benzoylbenzoic acid hydroxyl semicarbazone (2)*

After addition of the regents as described for compound 1 (method A), the mixture was heated under reflux for 1 h and then stirred at room temperature for 24 h. The mixture was concentrated by vacuum distillation and refrigerated for 1 h. The precipitate was filtered and recrystallized form methanol.

White powder (4.24 g, 72%): mp 230-231 °C; IR (KBr): 3000 (OH), 3160 (NH), 1670 (C=O); ^1^H NMR (DMSO-*d*_6_/500 MHz): *δ* 7.56-7.65 (m, 6H, Ar H), 7.68 (m, 1H, Ar H), 7.92 (m, 2H, Ar H), 8.36 (1H, s, Ar H), 12.88 (s, 1H); ^13^C NMR (CDCl_3_/125 MHz): *δ *160.8, 148.3, 135.4, 133.6, 131.8, 130.0, 129.5, 128.7, 127.1, 77.8, 77.5, 77.3, 40.5, 40.4, 40.2; Anal. Calcd for C_15_H_13_N_3_O_4_ (299.28): C, 60.20; H, 4.38; N, 14.04. Found: C, 60.29; H, 4.38; N, 14.00.

*2,4-Dihydroxybenzophenone hydroxyl semicarbazone (3)*

After addition of the regents as described for compound 1 (method A), the mixture was heated under reflux for 24 h and then stirred at room temperature for 48 h. The mixture was concentrated by vacuum distillation and refrigerated for 1 h. The precipitate was filtered and the title compound with acceptable purity was obtained.

Yellow powder (4.24 g, 74%): mp 184-186 °C; IR (KBr): 3100 (OH), 3200 (NH), 1634 (C=O); ^1^H NMR (CDCl_3_/500 MHz): *δ* 12.40 (s, 1H), 11.95 (s, 1H), 9.9 (s, 1H), 9.27 (s), 7.39 (m, 3H, Ar H), 7.13 (d, *J*=7.26, 2H, Ar H), 6.51 (d, *J*=8.78, 1H, Ar H), 6.13 (s, 1H, Ar H), 6.03 (dd, *J*=8.79, *J*=2.33, Ar H); Anal. Calcd for C_14_H_13_N_3_O_4_ (287.27): C, 58.53; H, 4.56; N, 14.63. Found: C, 58.40; H, 4.57; N, 14.65.

*4,4’-Dihydroxybenzophenone hydroxyl semicarbazone (4)*

The reaction was carried out as described for compound 1 (method A), except that an appropriate amount of molecular sieve was added to the reaction mixture. After heating the mixture under reflux for 24 h, stirring for further 48 h and typical workup as described earlier, the title compound was obtained.

Yellow powder (3.50 g, 60%): mp 141-144 °C; IR (KBr): 3200 (OH), 1630 (C=O); ^1^H NMR (CDCl_3_/500 MHz): *δ* 12.20 (s, 1H), 10.72 (s, 1H), 7.61 (m, 3H, Ar H), 7.54 (m, 2H, Ar H), 7.37 (d, *J*=8.66, 1H, Ar H), 6.38 (m, 2H, Ar H); Anal. Calcd for C_14_H_13_N_3_O_4_ (287.27): C, 58.53; H, 4.56; N, 14.63. Found: C, 58.60; H, 4.56; N, 14.69.

*Acetophenone hydroxysemicarbazone (5)*

After addition of the regents as described for compound 1 (method A), the mixture was heated under reflux for 1 h and then stirred at room temperature for 1 h. The precipitate thus formed was filtered and recrystallized form 2-butanol.

Yellow powder (2.90 g, 75%): mp 144-146 °C; IR (KBr): 3300 (NH), 3200 (OH), 1680 (C=O); ^1^H NMR (CDCl_3_/500 MHz): *δ* 8.61 (br s, 1H), 8.35 (br s, 1H), 7.63 (d, *J*=6.96, 2H, Ar H), 7.35 (m, 1H, Ar H), 7.29 (m, 2H, Ar H), 2.15 (s, 3H, C*H*_3_), 2.1 (s); Anal. Calcd for C_9_H_11_N_3_O_2_ (193.20): C, 55.95; H, 5.74; N, 21.75. Found: C, 55.87; H, 5.75; N, 21.71.

*Methoxyacetophenone hydroxyl semicarbazone (6)*

After addition of the regents as described for compound 1 (method A), the mixture was heated under reflux for 1 h and then stirred at room temperature for 2 h. The precipitate thus formed was filtered and the title compound with acceptable purity was obtained.

Light brown powder (3.25 g, 73%): mp 181-182.5 °C; IR (KBr): 3360, 3220, 2840, 1660; ^1^H NMR (CDCl_3_/500 MHz): Mixture of *E/Z* isomers *δ* 8.15 (s, 1H), 7.94 (d, *J*=8.55, 2H, Ar H), 7.65 (d, *J*=8.70, 2H, Ar H), 6.96 (d, *J*=8.86, 2H, Ar H), 6.90 (d, *J*=8.95, 2H, Ar H), 3.87 (s, 3H, OC*H*_3_), 3.85 (s, 3H, OC*H*_3_), 2.19 (s, 3H, C*H*_3_), 2.09 (s, 3H, C*H*_3_); Anal. Calcd for C_10_H_13_N_3_O_3_ (223.23): C, 53.80; H, 5.87; N, 18.82. Found: C, 53.91; H, 5.86; N, 18.79.

*In-vitro evaluation of antibacterial activity*

Compounds were assessed for their antibacterial activity by broth microdilution method. The strains used were *S. aureus *ATCC 6538, *E. Coli *ATCC 8439, *P. aeruginosa *ATCC 2097, *K. pneumonia* ATCC 10031 and *M. luteus *ATCC 9341. All the strains were cultured in Soybean Casein Digest Agar (SCDA) and after overnight incubation were diluted to 0.5 McFarland turbidity standards.

Different concentrations of the test compounds (10 µL of each) were added to 96 well plates. Then, 80 µL of Muller Hinton Broth (MHB) medium and 10 µL of microbial suspensions were added to each well. The final concentration of the microbial suspensions in each well was 1.5 × 10^7^ cfu/mL. The plates were sealed to minimize the evaporation of the medium and incubated at 35 °C for 24 h. The optical density of the wells was read at 580 nm using an ELISA reader spectrophotometer (TECAN-SP). The inhibitory concentration (IC) in each well was calculated by the following formula:

$\mathrm{IC}=\frac{\mathrm{ODc}-(\mathrm{ODa}-\mathrm{ODb})}{\mathrm{ODc}}$

Where ODa, ODb and ODc are the optical density of the solutions containing microorganisms and test compounds, only test compounds and only microorganisms respectively. IC_50_ was defined as the lowest concentration of the test compound in which the bacterial growth was completely inhibited. Amikacin and vancomycin were used as standard antibiotics. It is notable that each assay was performed as duplicates.

## Results and Discussion

*Chemistry*

**Scheme 1:**
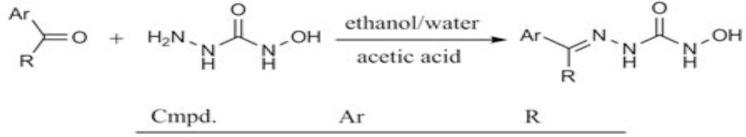
Synthesis of *N*-hydroxy semicarbazones 1-6

As disclosed in scheme 1, the final derivatives were prepared by a Schiff base formation reaction. The condensation between (thio) semicarbazide and aromatic ketones and aldehydes in the presence of an acidic catalyst often leads to stable crystalline products in good to excellent yields. However, in cases that (thio) semicarbazones of diaryl ketones are desired, a more challenging reaction could be anticipated and may need some modifications to force the reaction to proceed. This happens because the two *π*-donating aromatic systems make their adjacent carbonyl group less reactive towards different nucleophiles. In this project, the acetophenone derivatives 5 and 6 were prepared in good yields and the reaction time was as short as 2-3 h. However, for preparing compounds 1-4, for the reasons discussed above, some modifications were mandatory. For instance, synthesis of compound 1 in a microwave reactor proved to be more efficient than conventional method (see experimental). The synthesis yield for the other benzophenone derivatives was improved by increasing the reaction time and addition of molecular sieve to the reaction mixture.

**Figure 4:**
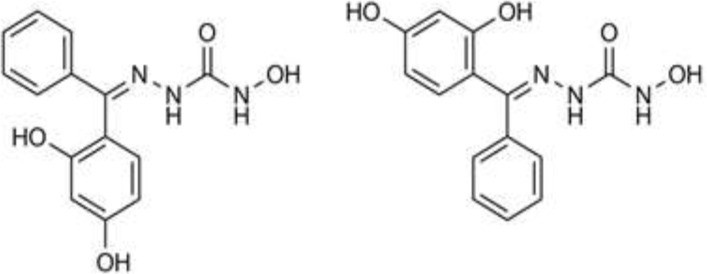
Structures of *E* (right) and *Z* (left) isomers of compound 3

The structure and purity of the synthesized derivatives were evaluated using different analytical methods such as IR, NMR and elemental analysis. In the IR spectra, stretch vibrations relating to O-H and N-H bonds in the hydroxysemicarbazone moiety was observed as broad weak bands in 3100-3350 region of spectra. In the carbonyl region of the spectra, the C=O stretch band appeared in 1630-1680 with medium to strong intensity. The relative complexity of ^1^H NMR spectra of the synthesized compounds reflects the fact that due to the rigidity of C=N bond, the two substituents on the ketimine carbon give rise to two different sets of hydrogens. This is observable in the ^1^H NMR spectra of compounds 1 and 4, where the aryl substituents on the ketimine carbon are the same (two phenyl rings in compound 1 and two *p*-hydroxyphenyl rings in compound 4). In contrast to benzophenone and 4,4’-dihydroxybenzophenone in which the hydrogens on one ring are chemical shift equivalent to their corresponding hydrogens on the other ring, in the ^1^H NMR spectra of compounds 1 and 4 the chemical shifts for the hydrogens of each ring is different from those on the other ring. As a result, two sets of double doublets are observed in the aromatic region of ^1^H NMR spectra of compound 4. *E/Z* isomerism as another outcome of C=N bond rigidity accounts for the complexity of the ^1^H NMR spectra of compounds 2, 3, 5 and 6 where two different substituents are present on the ketimine carbon. For instance, compound 3 could be assumed as two different isomers (Figure 4). The aryl substituents in each isomer “feel” different external magnetic fields and if both isomers are present in the sample, a complex ^1^H NMR spectra will not be surprising. Interestingly, the ^1^H NMR spectra of compound 6 indicates the presence of *E/Z* isomers with 1:1 proportion. Our findings are supported by some reports in the literature on the *E/Z* isomerism of hydrazone derivatives of diaryl ketones. For instance Rihardson *et al*. in their published work have obtained some benzoylpyridine thiosemicarbazone derivatives as a mixture of *E/Z* isomers (14). 

*Biological* *activity*

The synthesized derivatives were evaluated for their antibacterial activity against 6 different strains and the MIC values are listed in table 1. In addition to hydroxysemicarbazone derivatives 1-6, we were interested in bioactivity evaluation of compound 7. This compound has been reported to exhibit antitumor activity against some cancer cell lines (15). Since many antitumor derivatives have also been reported to possess antimicrobial activity (16-19), antimicrobial activity assessment of compound 7 as a previously established anticancer agent in order to evaluate its potential to be considered as a lead in antimicrobial drug discovery seems logical. Furthermore, since the hydroxysemicarbazone moiety is replaced by a phenylhydrazine derivative in compound 7, this derivative can be used to assess the role of hydroxysemicarbazone moiety in the tested derivatives. 

As it appears in table 1, compound 7 showed the highest activity against *P. aeruginosa *and *E. Coli *with IC_50_ values of 62.5 and 31.25 µg/mL. Among the substituted benzophenone derivatives, compound 2 with 2-carboxy substituent was the most active derivative especially against gram-negative strains such as *E. Coli*, *P. aeruginosa* and *K. pneumonia*. While the 4-methoxyacetophenone derivative 6 showed moderate activity against *E. Coli*, *P. aeruginosa*, the acetophenone derivative 5 did not show any activity at 1000 µg/mL concentration. 

Based on the analysis of the activity data, it is suggested that the presence of hydrophilic substituents on the aromatic ring is important for antibacterial activity. As evident from the activity data, 2-carboxy substituted benzophenone derivative 2 showed higher activity than the unsubstituted benzophenone derivative 1. Similarly, 4-methoxysubstituted acetophenone derivative 6 exhibited higher potency than its unsubstituted analog (compound 5). It is notable that compounds 2 and 5 demonstrated some selectivity through inhibition of gram-negative bacteria. This might be due to higher penetration of these hydrophilic derivatives inside the gram-negative bacteria through their hydrophilic porin channels which leads to availability of higher toxic concentrations of the tested derivatives inside the microorganism. Besides the hydroxysemicarbazones 1-6, phenylhydrazone derivatives 7 exhibited promising antibacterial activity which gives us a new opportunity to conduct more focused studies on the antibacterial activity of other phenylhydrazone derivatives of diaryl systems. 

**Table 1:** Antibacterial activity of the synthesized derivatives

**Compounds**	MIC (µg/mL)				
				
1	> 1000	500	250	250	1000
2	500	125	125	62.5	500
3	125	> 1000	500	> 1000	250
4	125	1000	500	1000	62.5
5	1000	1000	1000	1000	1000
6	500	500	250	125	500
7	500	125	62.5	31.25	500
Amikacin	4	0.5	2	1	2
Vancomycin	≤ 4	≤ 4	32	≤ 4	≤ 2
DMSO	+	+	+	+	+

## Conclusion

In this study, based on the pharmacophoric elements of active hydroxythiosemicarbazones and semicarbazones, a group of hydroxysemicarbazone derivatives were synthesized. The synthetic yields of the diaryl ketone derivatives improved by employing either microwave technique, increasing the reaction time or addition of molecular sieve. The derivatives were analyzed by different spectroscopic methods and among them, the ^1^H NMR data confirmed the formation of some derivatives as a mixture of *E/Z* isomers. The antibacterial activity of the synthesized derivatives was assessed against 6 pathogenic bacterial strains and compounds 2, 6 and 7 exhibited the highest activity. Notably, the activity of these derivatives was more pronounced against gram-negative bacteria. The facts that both compounds 2 and 7 possess hydrophilic substituents and selectivity over gram-negative bacteria, suggest their possible higher capability to pass through the porin channels available on the cell wall of the microorganism as a reasonable explanation for their antibacterial activity.

## References

[1] Kalinowski DS, Richardson DR (2007). Future of toxicologys–Iron chelators and differing modes of action and toxicity: The changing face of iron chelation therapy. Chem. Res. Toxicol.

[2] Dover LG, Alahari A, Gratraud P, Gomes JM, Bhowruth VE (2007). EthA, a common activator of thiocarbamide-containing drugs acting on different mycobacterial targets. Antimicrob. Agents. Chemother.

[3] Qian L, Ortiz de Montellano PR (2006). Oxidative activation of thiacetazone by the Mycobacterium tuberculosis flavin monooxygenase EtaA and human FMO1 and FMO3. Chem. Res. Toxicol.

[4] Laxmi SV, Rajitha B (2012). Synthesis and antimicrobial activity of newer indole semicarbazones. Med. Chem. Res.

[5] Sriram D, Yogeeswari P, Thirumurugan R (2004). Antitubercolous activity of some aryl semicarbazone derivatives. Bioorg. Med. Chem. Lett.

[6] Jafri L, Ansari LF, Jamil M, Kalsoom S, Qureishi S, Mirza B (2012). Microwave-assisted synthesis and bioevaluation of some semicarbazones. Chem. Biol. Drug Des.

[7] Chipeleme A, Gut J, Rosenthal PJ, Chibale K (2007). Synthesis and biological evaluation of phenolic Mannich bases of benzaldehyde and (thio)semicarbazone derivatives against the cysteine protease falcipain-2 and a chloroquine resistant strain of Plasmodium falciparum. Bioorg. Med. Chem.

[8] Sriram D, Yogeeswari P, Dhakla P, Senthilkumar P, Banerjee D (2007). N-Hydroxythiosemicarbazones: Synthesis and in-vitro antitubercular activity. Eur. J. Med. Chem.

[9] Haj Mohammad Ebrahim Tehrani K, Kobarfard F, Azerang P, Mehravar M, Soleimani Z, Ghavami G, Sardari S (2013). Synthesis and antimycobacterial activity of symmetric thiocarbohydrazone derivatives against Mycobacterium bovis BCG. Iran. J. Pharm. Res.

[10] Haj Mohammad Ebrahim Tehrani K, Sardari S, Mashayekhi V, Esfahani Zadeh M, Azerang P, Kobarfard F (2013). One pot synthesis and biological activity evaluation of novel Schiff bases derived from 2-hydrazinyl-1,3,4-thiadiazole. Chem. Pharm. Bull.

[11] Mashayekhi V, Haj Mohammad Ebrahim Tehrani K, Amidi S, Kobarfard F (2013). Synthesis of novel indole hydrazone derivatives and evaluation of their antiplatelet aggregation activity. Chem. Pharm. Bull.

[12] Haj Mohammad Ebrahim Tehrani K, Mashayekhi V, Azerang P, Minaei S, Sardari S, Kobarfard F Synthesis and antimycobacterial activity of some triazole derivatives – New route to functionalized triazolopyridazines. Iran. J. Pharm. Res.

[13] Ren S, Wang R, Komatsu K, Bonaz-Krause P, Zyrianov Y, McKenna CE, Csipke C, Tokes ZA, Lien EJ (2002). Synthesis, biological evaluation, and quantitative structure-activity relationship analysis of new Schiff bases of hydroxysemicarbazide as potential antitumor agents. J. Med. Chem.

[14] Debebe Z, Nekhai S, Ashenafi M, Lovejoy DB, Kalinowski DS, Gordeuk VR, Byrnes WM, Richardson DR, Karla PK (2012). Development of a sensitive HPLC method to measure in-vitro permeability of E- and Z-isomeric forms of thiosemicarbazones in Caco-2 monolayers. J. Chromatogr. B Analyt. Technol. Biomed. Life. Sci.

[15] Morgan LR, Thangaraj K, LeBlanc B, Rodgers A, Wolford LT, Hooper CL, Fan D, Jursic BS (2003). Design, synthesis, and anticancer properties of 4,4'-dihydroxybenzophenone-2,4-dinitrophenylhydrazone and analogues. J. Med. Chem.

[16] Mohebbi S, Shirazi FH, Sharifnia SH, Kobarfard F (2011). Introducing "synthesis route-based hit identification approach" as a tool in medicinal chemistry and its application in investigating the antiproliferative and antimicobial effects of 2-ainopyrimidine derivatives. Int. J. Drug Disc.

[17] Carta A, Sanna P, Gherardini L, Usai D, Zanetti S (2001). Novel functionalized pyrido[2,3-g]quinoxalinones as antibacterial, antifungal and anticancer agents. Il Farmaco.

[18] Al-Trawneh SA, Zahra JA, Kamal MR, El-Abadelah MM, Zani F, Incerti M, Cavazzoni A, Alfieri RR, Petronini PG, Vicini P (2010). Synthesis and biological evaluation of tetracyclic fluoroquinolones as antibacterial and anticancer agents. Bioorg. Med. Chem.

[19] Abd El Razik HA, Abdel Wahab AE (2011). Synthesis and biological evaluation of some novel fused pyrazolopyrimidines as potential anticancer and antimicrobial agents. Arch. Pharm.

